# Research progress on the treatment of diabetic nephropathy with leech and its active ingredients

**DOI:** 10.3389/fendo.2024.1296843

**Published:** 2024-01-26

**Authors:** Feng Tian, Xiang Yi, Feifei Yang, Yao Chen, Wenhui Zhu, Peng Liu, Shuju Li

**Affiliations:** ^1^ Renal Division, Department of Medicine, Heilongjiang Academy of Chinese Medicine Sciences, Harbin, China; ^2^ Shunyi Hospital, Beijing Hospital of Traditional Chinese Medicine, Beijing, China

**Keywords:** diabetic nephropathy, leech, hirudin, traditional herbal medicine, research progress

## Abstract

Diabetic nephropathy (DN) is a major microvascular complication of diabetes and a common cause of chronic kidney disease. There is currently a lack of effective treatments for DN, and the prognosis for patients remains poor. Hirudin, one of the primary active components derived from leeches, demonstrates anti-coagulant, anti-fibrotic, anti-thrombotic, and anti-inflammatory properties, exhibiting significant protective effects on the kidneys. In recent years, there has been a surge of interest in studying the potential benefits of hirudin, especially in its role in the management of DN. This article delves into the mechanisms by which hirudin contributes to the treatment of DN and its clinical efficacy.

## 1 Introduction

DN stands as the most prominent microvascular complication of diabetes, and it’s frequently the leading cause of death among diabetic patients. Globally, DN is the primary cause of end-stage renal disease (ESRD) (1). In China, with the increasing incidence of diabetes, the prevalence of DN is also on the rise (2).

Leeches have long been utilized in traditional Chinese medicine, demonstrating significant therapeutic efficacy in treating renal-related disorders. Modern pharmacological studies have identified that the principal component extracted from leeches is hirudin. This polypeptide, composed of 65-66 amino acid residues, serves as a natural thrombin inhibitor, showcasing properties like anticoagulation, antifibrotic, antithrombotic, and anti-inflammatory effects (3).

**Figure 1:**
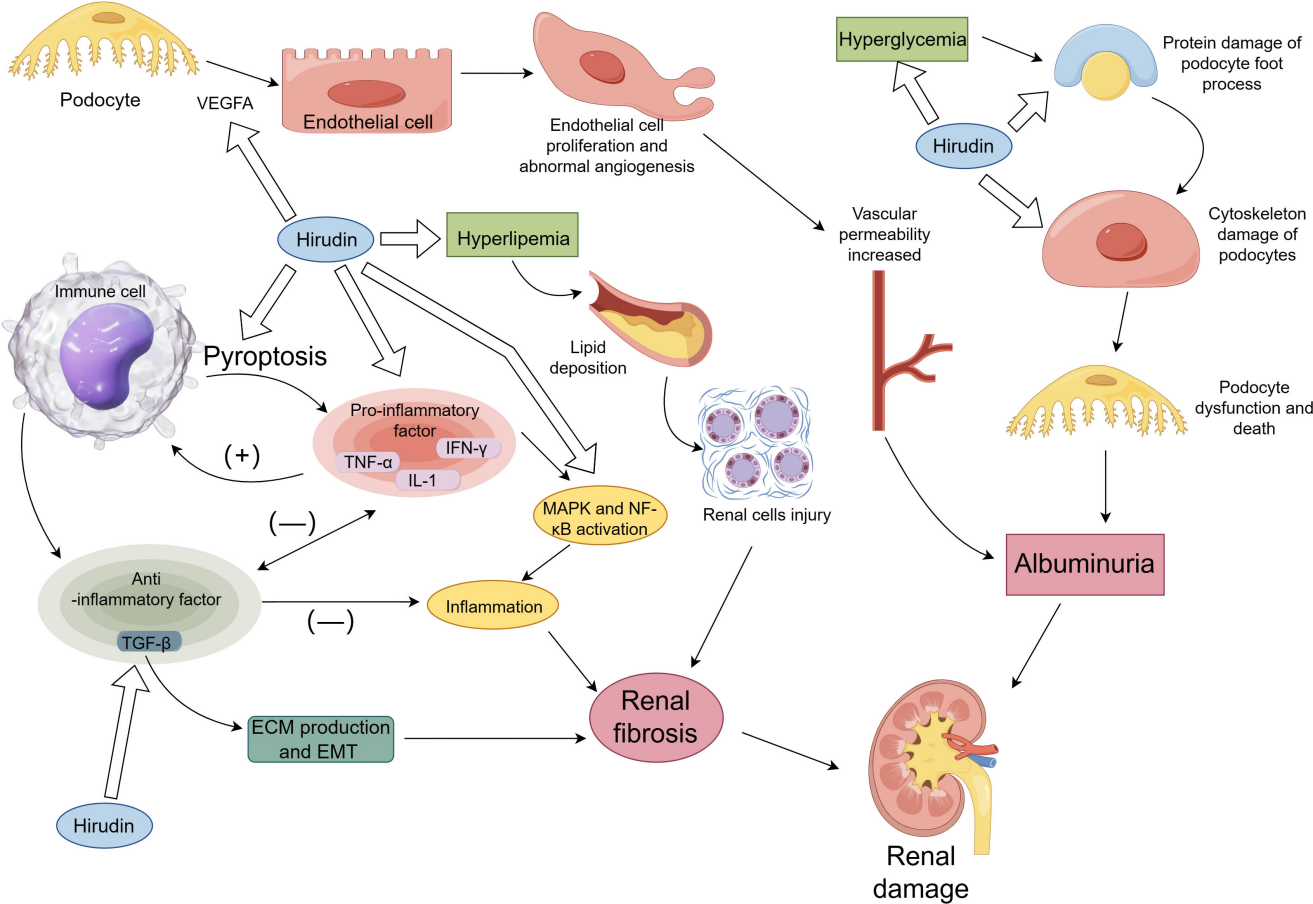
The mechanism of hirudin in DN. **Figure 1** briefly describes the mechanisms involved in renal damage and shows the renal protective effects of hirudin, including modulation of hyperglycemia and hyperlipidemia, inhibition of TGF-β-mediated renal fibrosis, inhibition of inflammation, pyroptosis, VEGF-mediated aberrant angiogenesis and protection of podocytes.

The mechanisms by which hirudin exerts its protective role on the kidneys are detailed in **Table 1** and **Figure 1**.

**Table 1:** Renal protective effect of hirudin.

Mechanism	Model	*In vivo/In vitro*	References
Metabolic Regulation	STZ-induced rats	*In vivo*	(4, 5)
Protection of Podocytes	db/db mice and HG-induced podocyte	*In vivo/In vitro*	(6)
	HG-induced podocyte	*In vitro*	(7)
	PAN-induced mice and PAN-induced podocyte	*In vivo/In vitro*	(8)
Inhibition of Inflammation	STZ-induced rats and HG-induced podocyte	*In vivo/In vitro*	(9)
Inhibition of Aberrant Angiogenesis	STZ-induced rats and HG-induced GEC	*In vivo/In vitro*	(10)
	STZ-induced rats and HG-induced HK-2 cells	*In vivo/In vitro*	(11)
	STZ-induced rats	*In vivo*	(12)
Inhibition of Pyroptosis	STZ-induced mice and HG/LPS and ATP-induced GECs, RTECs, and BMDMs	*In vivo/In vitro*	(13)
Inhibition of Renal Fibrosis	UUO rats	*In vivo*	(14)
	STZ-induced rats and HG-induced HK-2 cells	*In vivo/In vitro*	(11)
	UUO mice and TGF-β-induced HK-2 cells, IMCD3 cells and NRK-52E cells	*In vivo/In vitro*	(15)
	UUO mice and TGF-β-induced HK-2 cells	*In vivo/In vitro*	(16)

## 2 Study on the mechanism of hirudin on DN

The processes implicated in renal harm in DN and the associated processes of safeguarding renal function by hirudin are depicted in **Figure 1**. This section presents a comprehensive account of the hirudin experimental investigation to alleviate renal harm in **Figure 1**, addressing several factors, such as metabolic regulation, protection of podocytes, inhibition of inflammation, inhibition of aberrant angiogenesis, inhibition of pyroptosis and inhibition of renal fibrosis.

### 2.1 Metabolic regulation

Persistent hyperglycemia is a primary cause of DN. Prolonged poor glycemic control further aggravates the condition, making blood sugar regulation an essential approach to slow down DN’s progression. Hirudin has been shown to reduce blood sugar levels and glycated hemoglobin in rat models of DN induced by a high-fat and high-sugar diet combined with Streptozotocin (STZ). Furthermore, it offers protective effects on renal function (4).

Dyslipidemia is a crucial factor in the microvascular complications seen in diabetic patients. Lipid disorders can adversely affect the fibrinolytic system, leading to a heightened viscosity in blood, thereby increasing the formation of micro-thrombi. This, in turn, intensifies the ischemic and hypoxic conditions in the renal tissues (17). Furthermore, lipid accumulation predominantly takes place in the renal tubules, which has been associated with tubulointerstitial fibrosis. Such accumulations have deleterious effects on glomerular cells and podocytes (18). Managing lipid levels can reduce the risk of these complications. Hirudin has demonstrated its capability to lower total cholesterol, high-density lipoprotein, and other lipid metabolism markers, as well as hemorheological parameters in DN rat models induced by a high-fat and high-sugar diet combined with STZ (5).

**Figure 1** shows that hyperlipidemia and hyperglycemia lead to renal damage and that hirudin protects the kidneys by regulating lipid and glucose metabolism.

### 2.2 Protection of podocytes

A hallmark of DN is the development of proteinuria, a result of podocyte injury. The slit diaphragm protein of podocytes governs the permeability of the glomeruli. Damage or loss of the slit diaphragm leads to a restructuring of the podocyte cytoskeleton (19, 20). In the early stages of DN, the podocyte foot processes vanish, which is closely related to the cellular cytoskeletal remodeling (21). The apical surface of the podocyte is covered with a negatively charged polysaccharide-protein complex, including podocalyxin and glomerular epithelial protein 1 (GLEPP1) (22, 23). This complex is a vital component of the glomerular charge barrier. Damage to this charge barrier can also result in proteinuria. Hirudin safeguards the podocytes of db/db mice, maintaining the cellular cytoskeleton of podocytes. In podocyte induced by high glucose, hirudin inhibits the activity of RhoA, preserving the slit diaphragm proteins nephrin and podocin of foot processes (6). Another study indicated that hirudin increases the expression of podocalyxin and GLEPP1 in the apical region of podocytes under high glucose conditions, thus shielding podocytes and maintaining the integrity of the glomerular filtration barrier’s structure and function (7). In puromycin aminonucleoside (PAN) mouse models and PAN-induced podocyte models, hirudin protects the kidneys and prevents proteinuria by suppressing the transmission of the p38 MAPK signaling pathway, reducing endoplasmic reticulum stress in podocytes, and attenuating the damage to the cytoskeletal proteins of podocytes by PAN (8).

As shown in **Figure 1**, hirudin protects the kidney by inhibiting protein and cytoskeletal damage in podocytes.

### 2.3 Inhibition of inflammation

Inflammatory reactions play a pivotal role in the progression of DN. Pro-inflammatory cytokines, especially TNF-α, IL-1, and IL-6, are of particular importance. The presence of IL6 mRNA has been confirmed in renal biopsy specimens of DN patients, specifically in the glomeruli and interstitium (24). Levels of IL-6 in serum and urinary have emerged as potential markers for DN (25). They directly reflect the renal tissue inflammation status in DN (26). The inflammatory response results in the production of anti-inflammatory factors, predominantly including TGF-β (27). The anti-inflammatory effects of TGF-β and its pro-fibrotic effects are demonstrated in **Figure 1**. Upon tissue injury and concomitant inflammation, TGF-β is expressed in significant quantities, thereby restraining the inflammatory response and averting further tissue damage. However, excessive TGF-β expression leads to augmented ECM synthesis and consequent fibrosis in the tissue (28). Although TGF-β has the ability to inhibit the synthesis of inflammatory cytokines, there have been limited studies assessing its potential as an anti-inflammatory mediator for therapy (29). In the STZ induced diabetic rat model, both urinary and renal tissue expressions of TNF were found to be elevated (30). In HG-induced podocytes, hirudin can reduce the expression of pro-inflammatory cytokines (TNF-α, IL-1β, and IL-6) and the activation of p38 and NF-κB. In STZ-induced DN rat models, hirudin lowers renal indicators such as serum creatinine (Scr) and blood urea nitrogen (BUN) by reducing macrophage infiltration and inhibiting the activation of p38 and NF-κB (9). Another study found that hirudin modulates the NF-κB signaling pathway. By suppressing the activation of NF-κB, hirudin mitigates the inflammatory response, thereby improving renal fibrosis (14).

### 2.4 Inhibition of aberrant angiogenesis

A hallmark of DN is its aberrant angiogenesis, with vascular endothelial growth factor (VEGF) acting as the main mediator of this abnormal vascular growth. Changes in local VEGF concentrations and distributions within renal tissues are closely related to proteinuria onset and the severity of renal lesions in patients. In DN mouse models induced by STZ, overexpression of VEGF in podocyte vessels has been found to accelerate the progression of diabetic nephropathy (31). A significant correlation exists between circulating VEGF-A and serum Hypoxia inducible factor-1α(HIF-1α) levels, related to the pathogenesis of DN (32). Prolonged overexpression of HIF-1α may eventually promote organ fibrosis (33). Research indicates that the HIF-1α/VEGF pathway plays a role in the regulation of the extracellular matrix (ECM) (34). Studies show that hirudin can inhibit the migration of glomerular endothelial cells induced by high glucose (HG) and reduce the expression of angiogenesis-related proteins by suppressing the RhoA/p38/NF-kB pathway. In STZ-induced DN rat models, hirudin inhibited the expression of angiogenesis-related proteins VEGF and thrombomodulin-1, alleviating renal damage in rats (10). Hirudin significantly enhanced the activity of HG-induced HK-2 cells, reducing cell ECM expression by modulating the HIF-1α/VEGF pathway. In STZ-induced DN rats, hirudin reduced ECM deposition by adjusting the HIF-1α/VEGF pathway, thereby improving kidney function (11). Hirudin also managed to suppress the expression of VEGF and transforming growth factor β (TGF-β) in STZ-induced DN rats, offering renal protection (12). As shown in **Figure 1**, hirudin attenuated renal injury by inhibiting the VEGF pathway.

### 2.5 Inhibition of pyroptosis

Pyroptosis is an inflammatory form of programmed cell death, marked by the creation of pores in the plasma membrane due to gasdermin D (GSDMD) stimulation, resulting in cellular swelling, release of cellular contents (including pro-inflammatory factors like IL-1β and IL-18), and ultimately leading to cell death through the activation of inflammatory vesicles such as NLRP3 (35). Recent findings have underscored a significant linkage between pyroptosis and the pathogenesis of DN (36). Gasdermin D-mediated pyroptosis contributes to podocyte injury in mouse diabetic nephropathy (37). Research suggests that in glomerular endothelial cells and renal tubular epithelial cells induced by HG, as well as macrophages induced by lipopolysaccharide and Adenosine 5’-triphosphate, hirudin can reduce the expression of Gsdmd, thus inhibiting cell pyroptosis. In DN mouse models induced by STZ, hirudin moderates renal injury by regulating Irf2, subsequently inhibiting the expression of Gsdmd, IL-1β, and IL-18 (13). As shown in **Figure 1**, cellular pyroptosis can lead to the release of pro-inflammatory factors. Hirudin inhibits pyroptosis and reduces the release of pro-inflammatory factors.

### 2.6 Inhibition of renal fibrosis

Hirudin inhibits ECM expression in STZ-induced DN rats and HG-induced HK-2 cells by modulating the HIF-1α/VEGF pathway (11). In STZ-induced DN rats, hirudin inhibited the expression of TGF-β (12). The degree of tubulointerstitial fibrosis is closely related to the progression of DN. The TGF-β/Smad signaling and inflammatory responses play significant roles in fibrosis. In unilateral ureteral obstruction (UUO) rats model, hirudin significantly reduces ECM accumulation induced by UUO by modulating the expression of fibronectin, collagen III, and α-smooth muscle actin. It attenuates the inflammatory response by suppressing the NF-κB signaling, while concurrently inhibiting the TGF-β/Smad signaling and PAR1 to alleviate renal fibrosis (14). Hirudin can decrease the expression of inflammatory cytokines such as IL-1β, IL-6, and TNF-α in renal cells induced by TGF-β and diminish Epithelial–mesenchymal transition (EMT) and renal cell apoptosis. Moreover, hirudin suppresses the expression of inflammatory factors, fibrotic proteins, and ECM in UUO mice, thus counteracting renal fibrosis (15). In the HK-2 cells and renal tissues of UUO mice, hirudin weakens the upregulation of PAR1, S1PR2, and S1PR3 mediated by TGF-β and downregulates the S1P/S1PR1/S2PR1 signaling-mediated PAR3, diminishing EMT, fibrosis, and MCP-1 expression in HK-2 cells induced by TGF-β (16). The fibre-promoting effects of TGF-β are shown in **Figure 1** and hirudin inhibits expression of TGF-β.

## 3 Study of leeches and herbal prescriptions

In a model of STZ-induced DN in rats with a high-fat diet, leech lyophilised powder reduced serum levels of MDA, TNF-α, IL-1β, and MCP-1, and restored SOD activity. This was achieved primarily through inhibiting the expression of proteins associated with the JAK2/STAT1/STAT3 pathway (38). In a model of STZ-induced DN in rats receiving a diet high in sugar and fat, Chinese herbal granules (containing leeches) were found to increase the expression of podocyte α-actinin-4, Synaptopodin protein, and cleavage proteins podocin and CD2AP, while also reducing levels of 24-hour urinary protein (24h-Upro) in DN rats (39, 40). Patients with DN exhibit albuminuria, initially presenting as microalbuminuria and progressing into massive proteinuria and renal decompensation (41). As albuminuria is present throughout the course of DN and is both a consequence and contributor to renal injury, it is crucial to control albuminuria as a means of delaying DN progression to ESRD. Several clinical observations have shown the effectiveness of leeches and their active components in decreasing albuminuria. For example, Maixuekang capsules (containing hirudin) in combination with telmisartan have been shown to reduce the 24h-Upro in patients with DN (42). Hirudin capsules combined with ginkgolide have been found effective in reducing Scr, BUN, and 24h-Upro levels in DN patients (43). Yiqi Huoxue Buxue kidney formula, which contains hirudin, effectively lowers Scr, BUN, and microalbuminuria levels and notably improves clinical symptoms (44). Qihi Jiangtang capsules can regulate the expression of microcirculatory-related factors like nitric oxide and endothelin-1, and decrease BUN, Scr, and 24h-Upro levels (45). Shuxietong and Naoxuekang, both containing hirudin, can lower MALB in DN patients, improve coagulation function, and show no severe complications like gastrointestinal bleeding (46). No adverse reactions, including liver dysfunction and others, were observed in patients with chronic kidney disease who consumed large amounts of leech powder (9-12 g/d) (47). Similarly, patients with DN who were treated with leech medication did not experience any adverse reactions such as liver dysfunction (43). After DN progresses to ESRD, haemodialysis is the main treatment for ESRD. The recombinant hirudin structure is distinct from hirudin and is commonly used for anticoagulant therapy (48). Recombinant hirudin prevents thrombosis in experimental haemodialysis and is suitable for use as an anticoagulant in this extracorporeal circulation (49). Recombinant hirudin prevents thrombosis in ESRD patients during haemodialysis (50). And the studies have shown that anticoagulation with recombinant hirudin in critically ill patients on continuous hemodialysis can be performed (51).

## 4 Conclusion and perspectives

The pathogenesis of DN is intricate, involving a multitude of mechanisms. There is limited research on hirudin’s ability to regulate oxidative stress, inflammation, and pyroptosis. Specific mechanisms regarding its influence on glucose and lipid metabolism remain understudied. Given the evident clinical efficacy of hirudin, exploring how to develop drugs that offer better therapeutic effects for patients is a question that researchers should pursue in the future.

## Author contributions

FT: Writing – original draft. XY: Writing – original draft. FY: Writing – original draft. YC: Writing – original draft. WZ: Writing – original draft. PL: Writing – original draft, Writing – review & editing. SL: Writing – original draft, Writing – review & editing.
